# Slithering Toward Clarity: Snakes Shed New Light on the Evolution and Function of Sex Chromosomes

**DOI:** 10.1371/journal.pbio.1001644

**Published:** 2013-08-27

**Authors:** Mary Hoff

**Affiliations:** Freelance Science Writer, Stillwater, Minnesota, United States of America

What makes males males, and females females? For the majority of animals, the answer lies in the sex chromosomes, which come in two distinct types, with the combination in which they are doled out determining the gender of the offspring. Most other traits are determined by genes found on matched pairs of chromosomes (“autosomes”), one from each parent, which, through dominant and recessive relationships and other genomic sleights of hand, provide a rich palette of possible traits for natural selection to work on.

**Figure 1:**
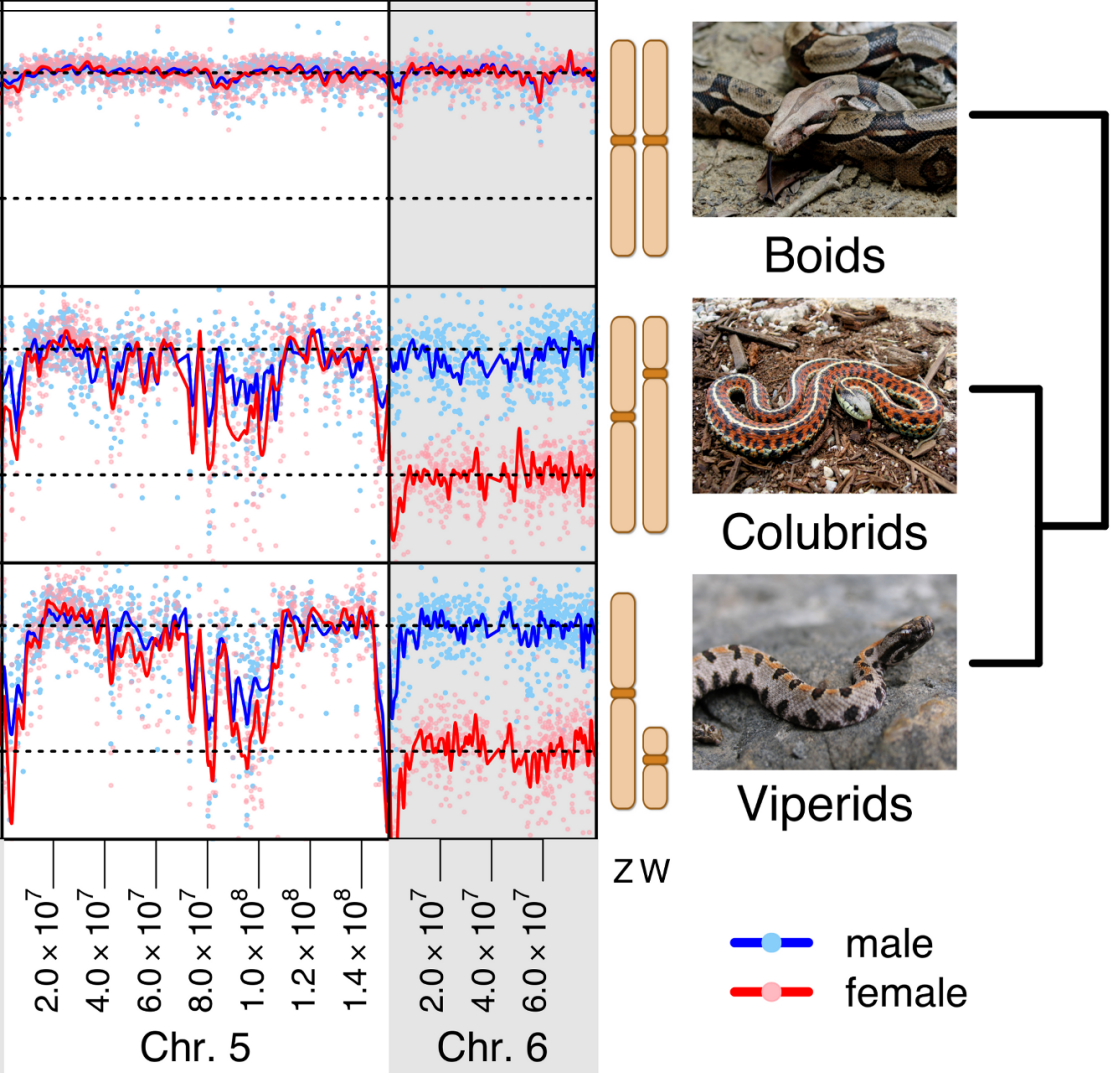
Sequences on the sex chromosomes (equivalent to lizard Chromosome 6) are present in half-quantities in the females of the colubrid garter snake and viperid rattle snake, reflecting degeneration of the W chromosomes. **Boid sex chromosomes and all snake autosomes (e.g., the equivalent of lizard Chromosome 5), on the other hand, don't differ in their representation between the sexes.**

Amazingly, considering the crucial role of sexual reproduction, sex chromosomes have evolved on multiple occasions. Genomic studies show that in each case the two sex chromosomes were once also a matched pair of autosomes, one of which has since degenerated over evolutionary time, with intriguing variations on the theme: In most mammals, presence of two undegraded (X) chromosomes produces a female (XX), while presence of a degenerate (Y) chromosome indicates a male (XY); in birds and reptiles, the matched set (ZZ) produces a male, while the unmatched (ZW) set produces a female. The degeneration process seems to start with a loss of the ability of the chromosome pair to exchange genes with each other (meiotic recombination), and proceeds with gradual reduction in expression, mutation accumulation, and eventual loss of genes on the Y or W. Similarities and differences between XY and ZW systems offer rich opportunity to explore the origins of sex chromosomes as well as the implications for how the traits they carry are expressed and shared from one generation to the next.

In search of a better understanding of the evolution and function of sex chromosomes, Doris Bachtrog, Beatriz Vicoso, J. J. Emerson, and colleagues took a gene's eye view of the sex chromosomes of three snake species in which the degradation of the W chromosome is at very different evolutionary stages: the boa, in which the two sex chromosomes appear identical under the microscope; a pygmy rattlesnake, in which the sex chromosomes appear quite different; and the garter snake, somewhere between the two. Examining the molecular genetics of the three chromosome sets in the context of each other and of the sex chromosomes of other species, the researchers uncovered valuable clues as to how sex chromosomes arise, how they change over evolutionary time, and how they contribute to making the organism what it is.

The researchers began by looking at the DNA sequences of the W and Z chromosomes from the three snake species. Comparing them to the fully mapped genome of a close relative, the anole lizard, they learned that the sex chromosomes in these snakes are analogous to chromosome 6 in the lizard (a nicely paired autosome), and identified which genes they carry. In the boa, where the Z and W chromosomes look alike, they found the genomic sequences of the two are also similar, and are still able to exchange genes through recombination. In the garter snake and rattlesnake, however, the researchers found that the two sex chromosomes had, in distinct evolutionary waves through time, lost the ability to exchange genetic material along much of their length. Because of similarities between the two species, they also concluded that the loss of recombining capabilities likely arose in a common ancestor before rattlesnakes and garter snakes evolutionarily diverged 50 million years ago. As expected from observations of other sex chromosome systems, the genes that remain on the W chromosome appear to be degenerate versions of those found on the Z.

Further examination of the three snake sex chromosome sets representing different stages of W chromosome degradation also shed light on how being sited on an unmatched set of chromosomes affects the evolution of individual genes. Comparing the three species' genomes at a molecular level, the researchers showed that Z genes evolve faster than those located on autosomes—in other words, evolution is male-driven in snakes, as has been previously observed in birds.

Finally, the team looked at the transcriptome—the RNA made by the genes—of the boa and rattlesnake to determine how degradation of genes on one of the sex chromosomes affects their expression. In the mammalian XY system, but not in the avian ZW system, the genes on the non-degraded chromosome make up for the degradation of their degraded counterparts by shutting down transcription on one of the X chromosomes in the female — a phenomenon known as global dosage compensation. The researchers found that in snakes, as in birds, rather than affecting the Z chromosome as a whole, dosage compensation appears to be rare and varies from gene to gene.

Overall, this comparative genome analysis adds a valuable second independent case of vertebrate ZW system evolution to the well-studied bird sex chromosomes. This will help us distinguish what's unusual about ZW chromosomes from what's unusual about birds.

**Vicoso B, Emerson JJ, Zektser Y, Mahajan S, Bachtrog D (2013) Comparative Sex Chromosome Genomics in Snakes: Differentiation, Evolutionary Strata, and Lack of Global Dosage Compensation. doi:10.1371/journal.pbio.1001643**

